# 18F-FDG PET/CT Did Not Increase the Risk of Cataract Occurrence in Oncology Patients: A Nationwide Population-Based Cohort Study

**DOI:** 10.3390/ijerph19137651

**Published:** 2022-06-22

**Authors:** Kai-Lun Cheng, Jing-Yang Huang, Jui-Hung Weng, Jeng-Yuan Chiou, Chyn-Tair Lan, Kwong-Chung Tung

**Affiliations:** 1 Department of Veterinary Medicine, National Chung Hsing University, 250 Kuo Kuang Road, Taichung 40227, Taiwan; chengkailun108@gmail.com; 2 Department of Medical Imaging, Chung Shan Medical University Hospital, 110 Jianguo North Road, Taichung 40201, Taiwan; 3 School of Medical Imaging and Radiological Sciences, Chung Shan Medical University, 110 Jianguo North Road, Taichung 40201, Taiwan; 4 Center for Health Data Science, Chung Shan Medical University Hospital, 110 Jianguo North Road, Taichung 40201, Taiwan; wchinyang@gmail.com; 5 Institute of Medicine, College of Medicine, Chung Shan Medical University, 110 Jianguo North Road, Taichung 40201, Taiwan; 6 Department of Nuclear Medicine, Chung Shan Medical University Hospital, 110 Jianguo North Road, Taichung 40201, Taiwan; cshy695@csh.org.tw; 7 School of Medicine, Chung Shan Medical University, 110 Jianguo North Road, Taichung 40201, Taiwan; 8 School of Health Policy and Management, Chung Shan Medical University, 110 Jianguo North Road, Taichung 40201, Taiwan; tom@csmu.edu.tw; 9 Department of Anatomy, Faculty of Medicine, Chung Shan Medical University, 110 Jianguo North Road, Taichung 40201, Taiwan; ctlan8899@gmail.com

**Keywords:** 18F-FDG PET/CT, radiation, cataract, oncology

## Abstract

This study aimed to evaluate the risk of cataract formation associated with radiation exposure from 18F-FDG PET/CT in oncology patients, using data from Taiwan’s National Health Insurance Research Database. The exposed group (Group E) consisted of oncology patients receiving 18F-FDG PET/CT within the first year of a cancer diagnosis. The comparison group (Group C) included subjects who had never been exposed to 18F-FDG PET/CT radiation and were propensity score-matched by date of enrolment, age, sex, cancer type, associated comorbidities, and CT utilization. Multiple Cox proportional hazard regression analysis was used to estimate the hazard ratio (HR) of cataract risk due to radiation exposure, while adjusting for potential confounding factors. A total of 703 patients and 1406 matched subjects were in Groups E and C, respectively. The incidence of cataract formation was not significantly higher among subjects in Group E (adjusted HR = 1.264; 95% confidence interval [CI] = 0.845–1.891). Our results revealed that 18F-FDG PET/CT was not a significant risk factor for developing cataracts in oncology patients.

## 1. Introduction

In addition to aging, ultraviolet radiation exposure, diabetes, obesity, alcohol consumption, smoking, and long-term use of corticosteroids, radiation exposure from medical imaging examination or procedures to the lens of the eye might be a risk factor for the development of cataracts in humans [1,2,3]. 18F-fluorodeoxyglucose (18F-FDG) PET/CT is a widely used imaging modality for the detection, staging, monitoring of therapy response, and restaging of oncology patients [4,5,6]. These patients receive not only radiation from the CT scan during examination but also radiation from the injected radiopharmaceutical, 18F-FDG. Therefore, side-effects on the radiosensitive lens from 18F-FDG PET/CT are a potential concern.

Although the literature shows that total absorbed radiation doses to the lens from CT scan and 18F-FDG are less than 8 mGy [7], which are lower than the single-dose threshold for cataract induction of 0.5 Gy (2012 International Commission on Radiation Protection guidelines [8]), an increased risk of cataract occurrence can still be found in individuals receiving a cumulative dose of <0.5 Gy [9]. Researchers have suggested various devices to protect medical personnel from occupational radiation exposure [10]; however, these devices are not commonly used by patients during radiation exposure examinations, which might induce an increased risk of iatrogenic radiation-induced cataract.

18F-FDG PET/CT is essential for oncology patients, and iatrogenic radiation-induced eye lens injuries in patients with advanced cancers might not be an important issue due to their potentially reduced life expectancy. However, many oncology patients can expect to live a normal lifespan after intensive treatment; therefore, 18F-FDG PET/CT examinations might pose a potential risk of cataract development and thus warrant further assessment. To date, no studies have investigated the association between radiation exposure from 18F-FDG PET/CT and the risk of cataract occurrence in oncology patients. Therefore, the purpose of this study was to estimate the association between cataract formation and radiation exposure from 18F-FDG PET/CT in a cohort of oncology patients registered in the Taiwan National Health Insurance Research Database (NHIRD).

## 2. Materials and Methods

### 2.1. Database

This retrospective cohort study was conducted according to the Declaration of Helsinki, and the protocol was approved by the Institutional Review Board of Chung Shan Medical University Hospital (project identification code: CS18054). The need for informed consent from participants was waived. The National Health Insurance (NHI) program covers approximately 99% of the Taiwanese population, and includes most nuclear medicine procedures, such as lymphoscintigraphy, bone scan, and 18F-FDG PET/CT scan for a total of 77 items [11]. For this study, data were retrieved from the Longitudinal Health Insurance Database 2000 (LHID 2000), a subset of the NHIRD, which contains one million beneficiaries randomly sampled from the original claims data of the 2000 Registry for Beneficiaries of the NHIRD. All registration and claim data of the LHID 2000 between 1 January 2005 and 31 December 2012 were collected for the subsequent analysis. Diseases and conditions studied were identified in the NHIRD based on the International Classification of Diseases, 9th Revision, Clinical Modification (ICD-9-CM). The NHIRD encrypts personal patient information to protect privacy, and it provides researchers with anonymous identification numbers associated with relevant claims information, including gender, date of birth, medical services received, and prescriptions. Therefore, patient consent is not required to access the NHIRD. Consequently, a longitudinal database and retrospective cohort study design were used to reveal the association between radiation exposure from 18F-FDG PET/CT and cataract occurrence.

### 2.2. Study Participants

**Figure 1:**
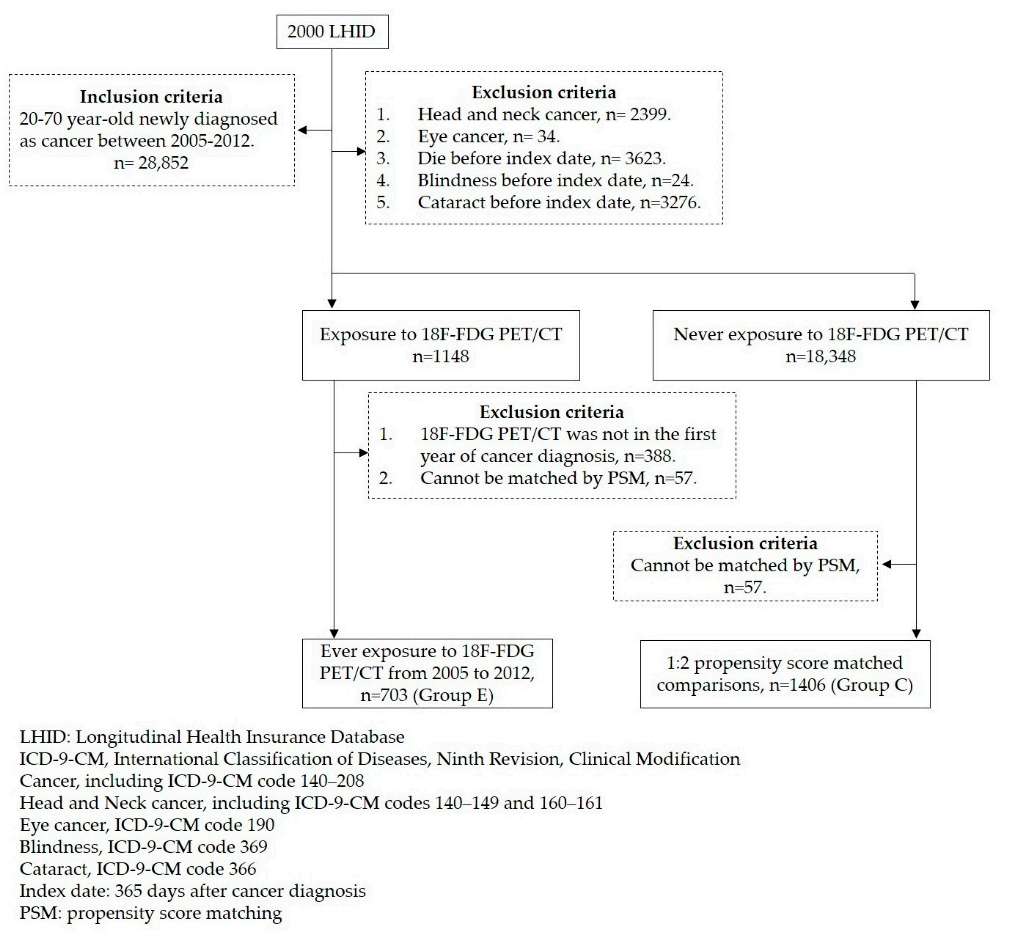
Study flowchart.

The exposed group (Group E) comprised patients newly diagnosed with cancers (defined as ICD-9-CM: 140–208) from 1 January 2005 to 31 December 2012. The index date was defined as 365 days after cancer diagnosis. The number of 18F-FDG PET/CT examinations during a period of 365 days before the index date was recorded. We excluded patients with missing demographic data and patients diagnosed with head and neck cancer (ICD-9-CM codes 140–149 and 160–161), eye cancer (ICD-9-CM codes 190), blindness (ICD-9-CM codes 369), cataract (ICD-9-CM code 366), or death before the index date (Figure 1).

### 2.3. Selected Controls

The control group (Group C) comprised patients who had never received 18F-FDG PET/CT examinations and that were retrieved from the LHID 2000 and matched at a propensity score ratio of 1:2 with patients enrolled in Group E. The index date of Group C was matched to that of Group E. Propensity score matching was used to control confounding factors, such as age, sex, cancer type, CT utilization, and pre-existing comorbidities, including hypertension (ICD-9-CM code 401–405); diabetes mellitus (ICD-9-CM code 250); chronic obstructive pulmonary disease (COPD; ICD-9-CM code 490–496); coronary artery disease (ICD-9-CM code 410–414); chronic renal disease (ICD-9-CM code 585–587); gout (ICD-9-CM code 274); rheumatoid arthritis (ICD-9-CM code 714.0); dementia (ICD-9-CM code 331.0, 290, 294); alcohol-related diseases (ICD-9-CM code 291, 303, 305.0, 357.5, 425.5, 535.3, 571.0, 571.1, 571.2, 571.3); depression (ICD-9-CM code 296, 300, 309, 311); sleep disorders (ICD-9-CM code 291.82, 292.85, 307.4, 327, 333.94, 347, 780.5, V69.4); obesity (ICD-9-CM code 278.00); hyperlipidemia (ICD-9-CM code 272.4); and atopic dermatitis (ICD-9-CM code 691).

### 2.4. Identification of Cataract

The endpoint of the study was the first occurrence of cataract. Subjects with a major event of cataract development were identified by at least two clinical visits [2,3] coded as ICD-9-CM 366 combined with prescribed therapies for cataract from the index date to the end of 2012. All participants were tracked from the index date to the date that a major event occurred, the date of censor, which included withdrawal from the NHIRD, or the end of the study (December 2012).

### 2.5. Statistical Analysis

All data were expressed as frequency (percentage). Next, categorical data were compared with the chi-square test. The time-to-event analysis was conducted to evaluate the relative risk of cataract incidence between Groups C and E. Poisson regression analysis was conducted to calculate the rate of cataract incidence and the associated 95% confidence interval (95% CI). Multivariate regression analysis using Cox proportional hazard regression analysis was employed to estimate the HR (95% CI) and thus evaluate whether 18F-FDG PET/CT exposure was an independent factor associated with increased risk of cataract, while adjusting for potential confounding factors. SAS version 9.4 was used for all statistical analyses, and a *p* value of <0.05 was considered statistically significant.

## 3. Results

As shown in Table 1, except for coronary artery disease, gout, rheumatoid arthritis, hyperlipidemia and the number of CT studies, no significant differences were found in the baseline demographic characteristics of age, sex, cancer type, comorbidities (i.e., hypertension, diabetes mellitus, COPD, chronic renal disease, dementia, alcohol-related disease, depression, sleep disorders, obesity and atopic dermatitis) between Groups C and E.

After adjusting for age, sex, cancer type, comorbidities, and CT utilization (Table 2), the adjusted hazard ratio (aHR) of 18F-FDG PET/CT exposure (Group E) was not found to be an independent risk factor for cataract occurrence (aHR = 1.264, 95% CI: 0.845–1.891, *p* = 0.255).

**Figure 2:**
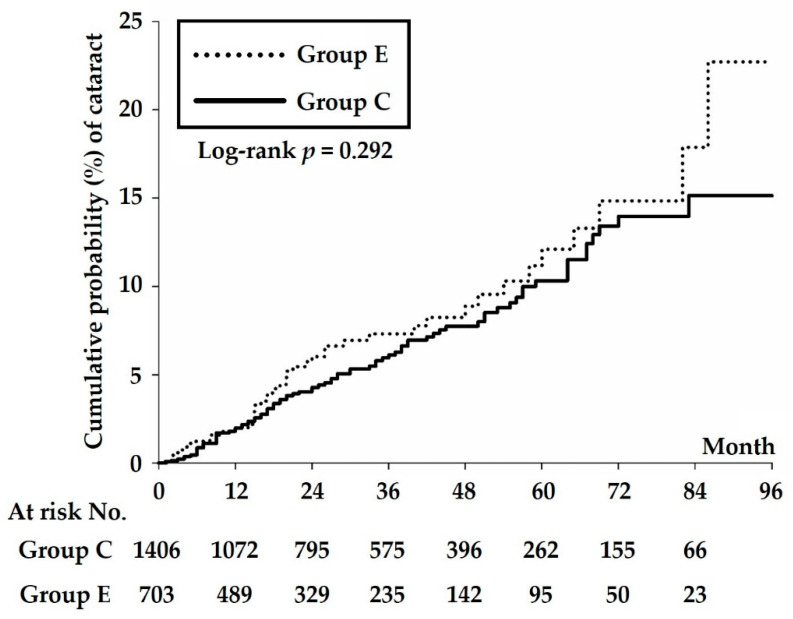
Kaplan–Meier curves of the cumulative probability of cataract occurrence in Group E and Group C. Group E did not reveal a significantly higher cataract occurrence (*p* = 0.292, log-rank test).

During follow up, Group E demonstrated a higher cumulative risk of cataract development, but the difference was not statistically significant (Figure 2).

We estimated the risk of cataract development using a multiple Cox proportional hazard model, and the results are presented in Table 3.

Contrary to 18F-FDG PET/CT exposure, which was not identified as a risk factor for cataract occurrence, the only independent risk factor for cataract occurrence was age (50–59 years-old, aHR: 6.437, 95% CI: 2.889–14.343, *p* < 0.0001; ≥60 years-old, aHR: 13.946, 95% CI: 6.225–31.244, *p* < 0.0001). To further elucidate the relationship among CT exposure, 18F-FDG PET/CT exposure, and cataract risk, the number of CT studies and the number of 18F-FDG PET/CT were further stratified into less than five, five or more, zero, one, and two or more, respectively, to compare the associated risk of cataract development (Table 4).

Except for the group of CT ≥ 5 and 18F-FDG PET/CT ≥ 2, the cataract incidence density showed a gradually increasing trend with the increasing number of CT and 18F-FDG PET/CT studies, although only the CT ≥ 5 and 18F-FDG PET/CT = 1 group showed significant differences (aHR = 2.380, 95% CI: 1.097–5.161).

## 4. Discussion

We used a comprehensive national population-based matched-cohort study to investigate the risk of cataract occurrence among oncology patients receiving 18F-FDG PET/CT. Our results suggest that 18F-FDG PET/CT is not associated with an increased risk of cataract occurrence in oncology patients. Despite the diversity and complexity of clinical situations among oncology patients, correction with a propensity score-matched model verified that 18F-FDG PET/CT did not increase the risk of cataract development (aHR = 1.264, 95% CI = 0.845–1.891). Additionally, the combination of at least one 18F-FDG PET/CT and ≥5 CT examinations within one year was positively correlated with the risk of cataract occurrence, but the finding was not statistically significant.

The lens has long been considered one of the most radiosensitive tissues in the human body. Radiation exposure increasing the risk of cataract occurrence has been well-documented in animal studies [12], as well as in other studies involving workers cleaning up after the Chernobyl nuclear accident [9] and atomic bomb survivors [13]. Although various types of protective eye equipment exist, patients are rarely provided with these during radiation examinations or procedures because of the potential generation of artifacts that could compromise image quality. However, this might increase the risk of iatrogenic-induced cataract. Several studies have investigated the association of cataract occurrence among patients exposed to medical radiation from different examinations [3,14] or interventional procedures [2]. The Taiwanese NHI program covers most reimbursements for oncology patients receiving 18F-FDG PET/CT and offers access to data for nationwide longitudinal studies. The present study is the first to investigate the association between 18F-FDG PET/CT and cataract occurrence in oncology patients.

The total 18F-FDG PET/CT dose is a combination of PET scan dose and CT scan dose. Organ doses from PET are estimated based on the injected 18F-FDG activity, while organ doses from CT are estimated based on different scanner types and protocol parameters [7,15,16,17,18]. The total effective dose of a whole-body 18F-FDG PET/CT study has been estimated to be approximately 25 mSv [16,17], and Paiva et al. found that the absorbed doses to the lens of the eye from CT and injected 18F-FDG were 4.71 mGy (effective dose: 0.06 mSv) and 2.94 mGy (effective dose: 0.05 mSv), respectively [7]. The 2012 International Commission on Radiation Protection guidelines revised the single-dose threshold for cataract induction from 5 to 0.5 Gy [8]. However, Worgul et al. found an increased cataract occurrence among 8607 Chernobyl clean-up workers, the majority of whom received a cumulative dose of <0.5 Gy [9]. Although the accumulated doses to the eye lens during 18F-FDG PET/CT reported by Paiva et al. might be significantly lower than the threshold for cataract formation, cataract occurrence is due to various causes and the potential risk associated with this examination still lacks evidence. The findings of the present retrospective study, which performed comparisons between an exposed group with an age-, sex-, cancer-type-, and comorbidity-matched control group, demonstrated that 18F-FDG PET/CT did not increase the risk of cataract occurrence in oncology patients.

In addition to 18F-FDG PET/CT, CT examination is another important imaging modality for oncology patients. The potential association between CT radiation exposure and cataract occurrence has been investigated in previous studies [3,14,19]. To delineate the confounding effect of CT examinations on cataract formation, subgroup analysis based on the number of CT studies was performed in the present study. Similar to 18F-FDG PET/CT, the number of CT studies was not found to be a predictor of cataract occurrence after multiple Cox regression analysis. The present result contradicts a report performed by Yuan et al. [3], which showed that repeated exposure to CT was significantly associated with increased risk of cataract formation. However, Yuan et al. only focused on the head and neck areas, whereas the present study included different types of cancers, which may explain this discrepancy. To delineate the potential combination effect, we compared the aHR of different numbers of 18F-FDG PET/CT and CT examinations in cataract occurrence. We demonstrated a trend, although not statistically significant, of an increased risk of cataract occurrence associated with increasing 18F-FDG PET/CT and CT frequency. However, there was an uncertainty in the estimated aHR, which may have resulted from the small number of patients in this group. The exact combination mechanism of action for 18F-FDG PET/CT and CT on cataract occurrence remains unclear. Therefore, further large sample studies or animal models are necessary for assessing this potential relationship.

Nonetheless, age was the only independent factor associated with an increased risk of cataract formation after multiple Cox regression in the present study, revealing that subjects aged 50–59 years (aHR = 6.437, 95% CI: 2.889–14.343) and ≥60 years (aHR = 13.946, 95% CI: 6.225–31.244) are at a higher risk of cataract development. These results are in accordance with the findings of two large studies on the association between age and cataract occurrence, i.e., the Beaver Dam Eye Study (BDES) conducted in the US [20] and the Blue Mountains Eye Study (BMES) conducted in Australia [21]. The increased risk of cataract occurrence with increasing age found in the present study is verified by BDES and BMES results. Many factors contribute to the progression of lens opacity, but increased age is the most frequently associated factor with cataract development. The findings of the present study are consistent with those of other studies [20,21,22], suggesting a natural aging process of the eyes.

The present study exhibited several limitations. First, the diagnosis of cataract was fully dependent on the ICD-9 codes; thus, cataract severity could not be clearly identified. Furthermore, we only included cases confirmed by two outpatient visits [2,3] so as to improve the diagnostic accuracy. However, sampled claims data from every hospital in Taiwan are randomly evaluated to verify their diagnostic accuracy, and false diagnostic reports yield severe penalties from NHIRD. Second, the NHIRD only provides information regarding the frequency of 18F-FDG PET/CT; however, different hospitals employ different PET/CT instrumentations and scan protocols. Therefore, we could not determine the exact radiation dose, PET/CT-related parameters, pharmacokinetic profile or biodistribution of 18F-FDG from every examination included in this study. Third, radiotherapy, which is particularly likely to induce cataracts [1], is commonly used in patients with head and neck cancer. In order to alleviate the possible confounding factors, large patient numbers with head and neck cancers were excluded from the case group. Fourth, medication use, such as corticosteroids, was not assessed in this study because miscellaneous drug prescriptions in Taiwan are difficult to extract from the NHIRD. Fifth, other personal risk factors related to cataractogenesis, such as smoking, overuse of the eyes, and lifestyle, cannot be obtained from the NHIRD. Sixth, since the study cohort only included oncology patients, a prospective study is needed to validate the conclusion within other areas of the general population, such as cancer screening. Finally, ethnic and racial disparities could affect cataract occurrence. Therefore, limited generalization of these results to different racial groups exists.

## 5. Conclusions

Our study indicated that exposure to 18F-FDG PET/CT did not pose an increased risk of cataract development among oncology patients. Consequently, 18F-FDG PET/CT is a safe imaging modality for these patients. Further prospective studies are necessary to validate these results.

## Figures and Tables

**Table 1:** Characteristics of oncology patients in Group E and Group C.

	Non-PET	PET	*p* Value
Sex			1.000
Female	776 (55.19%)	388 (55.19%)	
Male	630 (44.81%)	315 (44.81%)	
Age at index date			0.496
20–49	481 (34.21%)	245 (34.85%)	
50–59	557 (39.62%)	261 (37.13%)	
≥60	368 (26.17%)	197 (28.02%)	
Cancer type			1.000
Lung cancer	400 (28.45%)	200 (28.45%)	
Colorectal cancer	314 (22.33%)	157 (22.33%)	
Breast cancer	210 (14.94%)	105 (14.94%)	
Esophagus cancer	66 (4.69%)	33 (4.69%)	
Lymphoma	134 (9.53%)	67 (9.53%)	
Others *	282 (20.06%)	141 (20.06%)	
Co-morbidities			
Hypertension	381 (27.10%)	191 (27.17%)	0.972
Diabetes mellitus	206 (14.65%)	94 (13.37%)	0.428
COPD	178 (12.66%)	85 (12.09%)	0.709
Coronary artery disease	119 (8.46%)	34 (4.84%)	0.003
Chronic renal disease	76 (5.41%)	27 (3.84%)	0.116
Gout	90 (6.40%)	24 (3.41%)	0.004
Rheumatoid arthritis	20 (1.42%)	3 (0.43%)	0.038
Dementia	10 (0.71%)	8 (1.14%)	0.315
Alcohol-related diseases	24 (1.71%)	8 (1.14%)	0.314
Depression	205 (14.58%)	107 (15.22%)	0.696
Sleep disorders	254 (18.07%)	136 (19.35%)	0.475
Obesity	9 (0.64%)	2 (0.28%)	0.285
Hyperlipidemia:	263 (18.71%)	73 (10.38%)	<0.0001
Atopic dermatitis	20 (1.42%)	11 (1.56%)	0.798
No. of CT in the first year			<0.0001
0	690 (49.08%)	114 (16.22%)	
1 to 4	647 (46.02%)	486 (69.13%)	
≥5	69 (4.91%)	103 (14.65%)	

Data are shown in number (%) of patients. The *p*-values for comparisons between the two categorical groups were determined with the chi-square test. All chronic conditions were defined by administrative claims using International Classification of Diseases, Ninth Revision, Clinical Modification (ICD-9-CM) codes. Lung cancer, ICD-9-CM: 162. Colorectal cancer, ICD-9-CM: 153, 154. Breast cancer, ICD-9-CM: 174. Esophagus cancer, ICD-9-CM: 150. Lymphoma, ICD-9-CM: 200, 201, 202. * Others, including ICD-9-CM: 151–152, 155–159, 163–173, 175–189, 191–199, and 203–208. COPD, chronic obstructive pulmonary disease.

**Table 2:** Risk of cataract occurrence in oncology patients with PET exposure.

	Group C	Group E	*p* Value
N	1406	703	
Follow up person months	47,373	20,079	
Event of cataract	86	44	
Incidence density ^§^	18.15 (14.70–22.42)	21.91 (16.31–29.45)	0.310
Crude hazard ratio	Reference	1.276 (0.885–1.841)	0.192
Adjusted hazard ratio ^†^	Reference	1.264 (0.845–1.891)	0.255

^§^ Incidence density of new-onset cataract per 10,000 person-month. ^†^ Adjusted for sex, age group, cancer type, comorbidities, and CT utilization.

**Table 3:** Multiple-variable Cox regression for estimating the hazard ratios of cataract development.

	aHR (95% CI)	*p* Value
PET exposure		
No	Reference	
Yes	1.281 (0.854–1.921)	0.231
Sex		
Female	Reference	
Male	0.947 (0.639–1.401)	0.784
Age at index date		
20–49	Reference	
50–59	6.330 (2.835–14.135)	<0.0001
≥60	14.151 (6.312–31.728)	<0.0001
Cancer type		
Lung cancer,	Reference	
Colorectal cancer	1.000 (0.620–1.614)	0.998
Breast cancer	0.580 (0.276–1.218)	0.150
Esophagus cancer	0.787 (0.299–2.071)	0.627
Lymphoma	0.582 (0.254–1.333)	0.201
Others	1.001 (0.579–1.728)	0.999
Co-morbidities		
Hypertension	1.384 (0.936–2.048)	0.104
Diabetes mellitus	1.078 (0.679–1.712)	0.750
COPD	1.222 (0.733–2.038)	0.442
Coronary artery disease	1.212 (0.726–2.025)	0.462
Chronic renal disease	0.939 (0.481–1.832)	0.853
Gout	0.732 (0.352–1.520)	0.402
Rheumatoid arthritis	2.182 (0.662–7.184)	0.200
Dementia	1.091 (0.146–8.135)	0.932
Alcohol-related diseases	0.710 (0.096–5.240)	0.737
Depression	1.202 (0.751–1.926)	0.443
Sleep disorders	1.232 (0.785–1.935)	0.364
Obesity	2.005 (0.271–14.807)	0.495
Hyperlipidemia:	1.400 (0.905–2.166)	0.131
Atopic dermatitis	0.366 (0.050–2.661)	0.321
No. of CT in the first year		
0	Reference	
1 to 4	0.840 (0.549–1.285)	0.421
≥5	1.608 (0.830–3.114)	0.159

aHR, adjusted hazard ratio; CI, confidence interval; COPD, chronic obstructive pulmonary disease.

**Table 4:** The combination effect of CT and 18F-FDG PET/CT.

	Person-Months	Event of Cataract	Incidence Density ^§^	Adjusted HR ^†^
CT < 5 and PET = 0 (n = 1337)	45,999	81	17.61 (14.16–21.89)	Reference
CT < 5 and PET = 1 (n = 482)	13,957	26	18.63 (12.68–27.36)	1.116 (0.693–1.795)
CT < 5 and PET ≥ 2 (n = 118)	3448	9	26.10 (13.58–50.16)	2.029 (0.976–4.219)
CT ≥ 5 and PET = 0 (n = 69)	1374	5	36.39 (15.15–87.43)	1.460 (0.567–3.760)
CT ≥ 5 and PET = 1 (n = 82)	1646	8	48.60 (24.30–97.19)	2.380 (1.097–5.161)
CT ≥ 5 and PET ≥ 2 (n = 21)	1028	1	9.73 (1.37–69.06)	0.727 (0.098–5.403)

^§^ Incidence density of new-onset cataract, per 10,000 person-month. ^†^ Adjusted for sex, age group, cancer type and co-morbidities.

## Data Availability

Data are available from the NHIRD published by Taiwan National Health Insurance (NHI) Bureau. Due to legal restrictions imposed by the government of Taiwan in relation to the “Personal Information Protection Act”, data cannot be made publicly available. The Longitudinal Health Insurance Database 2000 (LHID 2000) was used for this study. For details of LHID 2000, please visit the website: https://nhird.nhri.org.tw/en/Data_Subsets.html#S3, accessed on 2 April 2022.
